# Leveraging community health workers for COVID-19 response in Democratic Republic of Congo, Nigeria, Senegal, and Uganda: roles, barriers, and facilitators

**DOI:** 10.1186/s12875-024-02531-0

**Published:** 2024-07-24

**Authors:** Noel Namuhani, Ziyada Babirye, Fred Monje, Mobolaji M. Salawu, Marc Bosonkie, Segun Bello, Steven N. Kabwama, Landry Egbende, Eniola A. Bamgboye, Andrew Tusubira, Yves Kashiya, Susan Kizito, Rotimi Felix Afolabi, Ayo S. Adebowale, Magbagbeola David Dairo, Issakha Diallo, Mamadou M. M. Leye, Youssou Ndiaye, Mane Fall, Oumar Bassoum, Ibrahima Seck, Olufunmilayo I. Fawole, Mala Ali Mapatano, Rawlance Ndejjo, Rhoda K. Wanyenze, Suzanne N. Kiwanuka

**Affiliations:** 1 Department of Health Policy, Planning and Management, School of Public Health, College of Health Sciences, Makerere University, P. BOX 7072, Kampala, Uganda; 2 Makerere University School of Public Health, Kampala, Uganda; 3 Department of Epidemiology and Medical Statistics, Faculty of Public Health, College of Medicine, University of Ibadan, Ibadan, Nigeria; 4 Department of Nutrition, School of Public Health, University of Kinshasa, Kinshasa, Democratic Republic of Congo; 5 Department of Community Health and Behavioural Sciences, Makerere University School of Public Health, Kampala, Uganda; 6 Department of Biostatistics and Epidemiology, Kinshasa School of Public Health, Kinshasa, Democratic Republic of the Congo; 7 Preventive Medicine and Public Health Department, Faculty of Medicine, Pharmacy and Dentistry, University Cheikh Anta Diop of Dakar, Dakar, Senegal; 8 Department of Disease Control and Environmental Health, School of Public Health, College of Health Sciences, Makerere University, Kampala, Uganda

**Keywords:** Community health workers, COVID-19, Roles, Barriers, Facilitators

## Abstract

**Background:**

The Corona Virus Disease 2019 (COVID-19) pandemic overwhelmed health systems and disrupted the delivery of health services globally. Community Health Workers (CHWs) play a critical role in linking communities to health systems, supporting the prevention and control of diseases in many low- and middle-income countries. However, their roles, barriers, and facilitators in the response and control of the COVID-19 pandemic have not been well documented. We described the roles of CHWs in the COVID-19 response, including the barriers and facilitators.

**Methods:**

A cross-sectional study design was used to assess the COVID-19 response in the Democratic Republic of Congo (DRC), Nigeria, Senegal, and Uganda. This involved 110 key informant interviews with policymakers, health facility managers, district health managers, and CHWs to understand the role of CHWs in the COVID 19 response, selected purposively. The total sample size was based on information saturation in each of the countries. A document review on the COVID-19 response was also conducted. We searched Google, Google Scholar, and PubMed for published and grey literature. Data from the selected documents were extracted into a Google master matrix in MS Excel and analyzed thematically.

**Results:**

In COVID-19 Control, CHWs supported community-based surveillance, contact tracing, risk communication, community mobilization, and home-based care. To support the continuity of other non-COVID-19 services, the CHWs conducted community mobilization, sensitizations, outreaches, referrals, and patient follow-ups. CHWs were challenged by movement restrictions, especially in the initial stages of the lockdown, inadequate PPE, increased workload, low allowances, and motivation. CHW were facilitated by trainings, the development of guidelines, development partners’ support/funding, and the provision of personal protective equipment (PPE) and tools.

**Conclusion:**

CHWs supported both the COVID-19 control and continuity of non-COVID-19 health care during the COVID-19 pandemic. CHWs are a critical resource that must be adequately supported to build resilient health systems.

**Supplementary Information:**

The online version contains supplementary material available at 10.1186/s12875-024-02531-0.

## Background

The Corona Virus Disease 2019 (COVID-19) pandemic, which was declared on March 11, 2020, by the World Health Organization (WHO) [1], had negative effects on health systems worldwide. Health systems were overwhelmed in a bid to contain the spread of the virus and, at the same time, maintain the delivery of non-COVID-19 health services, especially in low-income countries [2]. This was further exacerbated by the inadequate health workforce in most African countries [3]. According to the WHO, about 94% of the countries had a disruption in at least one Essential Health Service (EHS), and one-third of all countries had disruptions in more than half of their EHS in 2021 [2]. These disruptions were attributed to the COVID-19 mitigation measures, including lockdowns, movement restrictions, suspension of public gatherings, and closure of health facilities. The measures made clients and health workers unable to access health facilities for services [4].

The disruption in continuity of non-COVID-19 services necessitated the adoption coping mechanisms. These included enacting policies and guidelines, increasing awareness campaigns on the availability of non-COVID-19 services, conducting outreaches, task sharing, redistribution of supplies and commodities, and the use of CHWs, who play a critical role in linking communities to health systems, supporting disease prevention and control efforts in many Low- and Middle-Income Countries (LMICs) as they are closer to the community and are trusted by community members [5, 6]. CHWs usually have no formal profession but are trained to provide health services to the community [5]. They often take on different names in different countries, such as Relais Communautaires (RECOs) in the DRC, Voluntary Health Workers (VHWs) in Nigeria, Community Health Providers (CHPs) in Senegal, and Village Health Team (VHT) in Uganda.

CHWs have supported the response to previous epidemics. For instance, CHWs were involved in Liberia’s response to the Ebola outbreak [7] and supported Ebola preparedness activities in Côte d’Ivoire during the same outbreak [8]. Throughout the 2014–2016 Ebola outbreak in West Africa, CHWs participated in contact tracing and case finding and referred probable Ebola cases to treatment centers [6, 7]. During COVID-19, CHWs played an instrumental role in most health systems across various countries. For example, in Uganda, CHW supported community-based surveillance, and in Senegal, they supported community delivery of ARVs for HIV patients, patient follow-up in the DRC, and awareness creation in Nigeria. However, the role and contributions of CHWs to the COVID-19 response across the different contexts within Sub-Saharan countries, are scantily documented. We aimed to document the key roles played by the CHWs in the COVID-19 response and the barriers and facilitators of their work in the DRC, Nigeria, Senegal, and Uganda. This will guide community health systems strengthening initiatives and building resilient health systems for pandemics.

## Methods

### Study area

This work is part of a multi-country project that was conducted in the DRC, Nigeria, Senegal, and Uganda as an assessment of the COVID-19 response. These countries were selected purposively due to their past experience with epidemics but also to represent Anglophone and Francophone countries.

### Community health system contexts

#### Democratic Republic of Congo (DRC)

In DRC, the health system is decentralized and operates across three levels including the national level, the provincial level and the health zone level. The health zone level comprises a constellation of around 20 health areas (HA). Each area operates one health center to which are linked several community health workers known as relais communautaires (RECO). A health development committee is organized in each health area comprising volunteer members of the community who are responsible for co-management of the health center with health providers [9].

#### Nigeria

The National Health System is decentralized into three tiers structures with responsibilities at federal, state, and local government levels. The Federal government handles the tertiary healthcare, the state oversees the secondary healthcare, while the local government takes responsibility of the primary healthcare. Nigeria is estimated to have about 75,000 Volunteer Village Health workers.

#### Senegal

Senegal’s health care system operates with a three-tiered structure. The three tiers include; heath Posts which are the lowest level and most available form of health care in Senegal; health Centers which are more scarce than health posts, but have better medical technology available and the regional Hospitals which are few and far in between but with most medical technology and the best ability to handle all sorts of patients [10]. There are also University and private care providers, but these are uncommon.

#### Uganda

Uganda has a decentralized health systems with VHTs being the lowest level at the community level. The VHTs program was established in 2001 mainly to link communities with health services. It is estimated that each village has 4 VHT members and these support mobilizing communities for better health services, conducting home visits, managing malaria, diarrhea, and pneumonia among children under five years, distributing health commodities, and conducting referrals to health facilities. Uganda is estimated to have 179,000 Village health team members [11].

### Study design

This was a cross-sectional qualitative study that involved literature reviews and key informant interviews on the roles of CHWs during the COVID-19 response, barriers that hindered CHWs from supporting the COVID-19 response, and what facilitated CHWs to support the COVID-19 response across the four countries.

### Data collection

A document review on the roles of CHWs in COVID-19 was followed by conducting key informant interviews during the period of November 2020 to March 2021 across the four countries.

### Document review

We searched Google, Google Scholar, and PubMed from November 2020 to March 2021 for documents that reported on COVID-19 Response using the following search terms: “COVID-19”, “Response”, “barriers”, “facilitators”, “COVID-19 Response”, “Village health teams”, “community health workers”, “Volunteer Village Health workers”, “Community health providers”, “Relais communautaires”. We also checked the websites of organizations involved in the response, such as the United Nations Populations Fund (UNFPA), the Ministry of Health (MOH), the United States Agency for International Development (USAID), and the United Nations Children Fund (UNICEF), for additional documents. The documents that fulfilled the inclusion criteria were reviewed. We extracted information from documents in the four countries with elements of the COVID-19 response highlighting the role of community health workers. Documents that did not cover the study countries were excluded.

The documents reviewed included COVID-19 response plans, reports, website guidelines, meeting minutes, and presentations for the DRC, Nigeria, Senegal, and Uganda. We also searched peer-reviewed and non-peer-reviewed publications regarding CHWs and the COVID-19 response in the four countries.

#### Key informant interviews

Key informant interviews were conducted to describe the roles of CHWs during the COVID-19 response and the facilitators and barriers they encountered. We developed an interview guide based on the study objectives (the tool is attached as a supplement). The respondents were selected from the national, sub-national, and village levels within selected communities in the DRC, Nigeria, Senegal, and Uganda. The 110 key informants included 20 health managers, including nurses, clinical officers who supervise the CHWs, eight ministries of health, and 29 community health workers. We conducted 22 interviews in the DRC, 32 in Nigeria, 21 in Senegal, and 35 in Uganda. Most (seventy four) of the respondents were female, with an average age of 38 years.

### Data management and analysis

All key informant interviews were voice-recorded and later transcribed verbatim. The transcripts were proofread by researchers to check the quality of the transcriptions. Identifiers were removed, and data for each key informant was stored separately. A thematic analysis approach was used [12]. Transcripts were then used to conduct an Excel-based, thematic matrix analysis. The matrix included deductive themes of inquiry, including the roles of CHWs in supporting the continuity of essential health services, COVID-19 control, barriers they faced, and facilitators of their work (Table 1). The data was summarized into a matrix, reviewed, and compared to the transcript to ensure inter-coder reliability. Data on each code were then aggregated by participant category to document the experiences of each participant group. Relevant quotes were included in the matrix to support the key findings; a few typical quotes are part of the study results section of this paper.

## Results

This section presents findings along four major themes: (1) Roles of CHWs in COVID-19 control; (2) Roles of CHWs in supporting continuity of access to health care; (3) Barriers faced by CHWs in supporting COVID-19 response; and (4) Facilitators of CHWs in supporting COVID-19 response. The table below summarizes the themes and sub-themes that emerged from the study (Table 1).

**Table 1:** A summary of themes and subthemes

Themes	Subthemes
COVID − 19 control	• Community-based surveillance • Contact tracing • Risk communication • Community mobilizations • Supporting home based care and quarantine
Continuity of access to Healthcare	• Community mobilization • Sensitizations • Task shifting • Outreaches • Referring patients • Conducting follow ups
Barriers	• Transport restrictions • Inadequate PPEs • Inadequate Lack of tools such as reporting forms • Lack of community engagement strategy and guidelines • Increased workload • Low allowances and motivation
Facilitators	• Capacity building • Development of strategies and guidelines • Development partners support/funding • Provision of PPEs • Mobile phones, • Provision of passes • Community will • Availability of community structures such as community radios

### Roles of community health workers in supporting COVID-19 control

The CHWs supported COVID-19 control through community-based surveillance, contact tracing, risk communication, community mobilization, and supporting home-based care and quarantine. The roles played by CHWs in supporting COVID-19 are described below.

#### Community-based surveillance

CHWs supported the community-based surveillance systems, especially in Uganda and the DRC, which were critical and effective in detecting COVID-19 cases. For instance, in Rakai District in Uganda, VHTs detected the first four COVID-19 cases in the district. These supported screening for COVID-19 symptoms and an active search of suspected cases. In the DRC, one of the key informants noted;

*“CHWs conducted active search of suspected cases in the community*,* and closely monitored contacts through home visits conducted during the 14 days of quarantine. During follow up*,* they checked for COVID-19 symptoms and arranged to test those that developed symptoms”***(Policy maker**,** DRC)**.

One of the district managers in Uganda also emphasized that:

*“The district ensured that each health facility and village had a trained*,* active VHT who was empowered to report any alerts using an integrated electronic surveillance reporting system by sending the message to 6767*,* for follow-up and action. These were supervised weekly by the district and sub county surveillance focal persons”* (**Manager**,** Uganda**).

#### Risk communication

Since the CHWs are the first point of contact, they delivered COVID-19 preventive messages to families and individuals within their communities in all four countries. They used community radios with loudspeakers to sensitize the community about COVID-19 prevention. CHWs also supported efforts to address misinformation about COVID-19 among the community. This was enabled by the fact that CHWs are people within the community who understand the best way to relate with the masses in a culturally appropriate context.

#### Contact tracing

Across the four countries, CHWs conducted contact tracing and notification of potential infections to district health teams. For instance, in the community-based surveillance in the DRC, Nigeria and Uganda, the CHWs working with the community actively searched for COVID-19 contacts and cases.

#### Community mobilization

CHWs supported community engagement and mobilization of the community to take up COVID-19 preventive measures, including hand hygiene. They also supported mobilizing the community for vaccine uptake. Across the four countries, CHWs conducted community mobilizations through visiting households, using community radios, and conducting outreaches.

#### Follow-up of patients

CHWs were also involved in following up on COVID-19 patients, monitoring any signs and responses to treatment, especially those who were in isolation and receiving home-based care. For example, in Uganda, VHTs visited households and regularly contacted the patients through phone calls to check on them and monitor any emerging signs and symptoms.

### Roles of community health workers in supporting continuity of access to healthcare

CHWs in the DRC, Nigeria, Senegal, and Uganda supported the continuity of access to other non-COVID-19 services. To support this, CHWs conducted community mobilization for services, sensitizations, task sharing, offered services through outreaches, referred patients, and followed up on clients. The roles played by CHWs in supporting continuity of access to health care are described below:

#### Community mobilization

In all the four countries, CHWs played a key role in mobilizing the community to access services at the health facilities. During lockdowns, most community members were afraid of accessing health facilities because of the fear to be infected with COVID-19. Some community members also believed that health facilities were closed due to the lockdown. CHWs passed on messages about the availability of services and dispelled the fear among community members about accessing Health facilities. The community mobilization was done using megaphones, community radios, and home visits. One of the key informants noted that.

*“CHWs mobilized HIV + clients in their communities so that they would meet at a central place such as a school or church or nearby facility to access their pills.”***(Health facility manager - Uganda)**.

#### Sensitization

Most key informants in the DRC, Nigeria, Senegal, and Uganda reported that CHWs conducted sensitization about family planning, nutrition, malaria, immunizations, antenatal care (ANC) services, sanitation, and integrated community case management services for children under five.

*“…awareness-raising campaigns on various health domains including Reproductive Maternal Neonatal Adolescent and Child Health*,* HIV*,* TB*,* Malaria resumed and were mainly done by the CHWs under close supervision of the chief nurse of the health area and the Physician chief of the health zone.”****(Policy maker – DRC)***.

*“I would say one of the innovations employed by health facilities was using community health workers to conduct health talks about preventable diseases like Malaria*,* sanitation related disease and COVID-19.”***(Health worker - Nigeria)**.

#### Offering services and task sharing

Across the four countries, there was task sharing and the assignment of some service provision roles to community health workers. In Uganda, half of the VHTs interviewed directly offered services for family planning, delivered ART services to HIV patients in the community, and distributed antibiotics and anti-malarial drugs to children under five years old. The VHTs also supported indoor residual spraying in northern Uganda. In the DRC, CHWs supported screening and treatment of minor conditions such as malaria, diarrhea, and pneumonia. In Nigeria, VHWs, together with traditional birth attendants, were trained to attend to deliveries at home in addition to antenatal and postnatal care. In Senegal, CHPs supported TB, HIV, and hypertensive patients by delivering medicines to them.

*“Community health workers supported Community delivery of ARVs and TB products*,* and distribution of Nutritional support in the form of food kits to key populations”***(Health worker - Senegal).**

#### Referring patients

In Uganda and Senegal, key informants reported that CHWs played a key role in referring patients to health facilities for care, and for some, working with local council chairpersons, they provided travel documents during lockdowns. For instance, CHPs referred patients from epidemic treatment centers (CTE) to other non-CTE sites to obtain their ARVs and TB medicines in Senegal. One of the VHTs in Uganda noted;

*“…I would tell them that if your child has a fever*,* go to the hospital very early in the morning… So I also gave them referral forms so that they could move easily to the hospital… And even if anybody went to the hospital and told the doctor that they were sent by the VHT they could be handled well. It helped us to get the children in the community to come and get services from the hospital on time.”* (**Community Health Worker** - Uganda).

#### Follow-up of patients

In all countries, CHWs followed up with clients to assess treatment outcomes and side effects through home visits. They followed up to monitor child growth and immunization, TB and HIV patients, diabetic and hypertensive patients.

*“…the TB Program recommended to the health zone management team to increase the number of CHWs for carrying out community Directly Observed Treatment i.e*,* the CHWs were trained to follow-up patients in households for providing TB treatment and ensuring they complied with the barriers measures for COVID-19.”* (**Program manager -DRC)**.

*“…as a VHT… I conducted home visits*,* sometimes I made phone calls to follow up patients and find out whether she/he accessed medicines*,* having any side effects*,* how well they are.” (***Community Health Worker -Uganda**).

### Barriers to community health worker activities during the COVID-19 response

The community health workers in all four countries faced challenges during the COVID-19 response. These included movement restrictions, especially in the initial stages of the lockdown due to the ban on public transport. There were also inadequate PPE, a lack of community engagement strategy and guidelines at the start of the pandemic, increased workload as the pandemic progressed, and low allowances, which demotivated some of them to continue providing services.

#### Transport restrictions

Across all four countries, lockdowns and the ban of public transport and movements were implemented. This limited CHWs’ ability to visit health facilities and communities, which limited home visits, outreaches, and follow-up with patients. This hindered CHW service delivery. In Uganda, transport restrictions led to increased transport fares, resulting in VHTs incurring more expenses, which further constrained their movement in the community. It also made it difficult for VHTs to follow up on TB patients, HIV, pregnant women, and sick children in communities.

#### Inadequate PPE and tools

CHWs were also challenged by inadequate PPE, including masks and gloves, and forms for registering and following up with patients. The inadequate supply of masks, gloves, and gumboots compromised their safety during the COVID-19 response efforts and provision of essential services. The PPE shortages were attributed to global PPE shortages and exorbitant prices, which made PPE unaffordable and accessible to most African countries.

#### Guidelines

At the start of the pandemic, Uganda lacked a community engagement strategy and guidelines on how the VHTs could support the response. The VHTs were not engaged in the response at the early stages. In particular, it was not clear how services would be delivered amidst restrictions on gatherings and physical distancing, as well as the wider travel and movement restrictions. This affected VHTs’ ability to access health facilities and some distant households.

#### Work load

As the pandemic progressed, the work load of the CHWs increased, and they worked longer hours. Additionally, the CHWs had to balance the additional work with domestic responsibilities, which exhausted and stressed some of them.

“*Oh my experience … it wasn’t funny it was.. ..draining physically and emotionally. It was a stressful period” (VHW Nigeria)*.

#### Low motivation and poor remuneration

Across all countries, respondents noted low motivation due to low pay as a barrier to supporting the COVID-19 response. In the DRC, the RECOs in Kinshasa went on strike because they were not given the promised remuneration. In Uganda, the remuneration was little, and it was paid late, which demotivated the RECOs.

### Facilitators of community health worker activities during the COVID-19 response

To strengthen community health systems to be able to support the COVID-19 response, several supporting activities were conducted, including training, the development of guidelines, development partners’ support and funding, the provision of personal protective equipment (PPE), mobile phones, the provision of passes, and the community will. These were mainly provided by the government, implementing partners, donors, and the community. The facilitators that enhanced CHWs activities during COVUD-19 response are elaborated below:

#### Training

In all countries, with support mainly from development partners, CHWs were trained on the maintenance of essential health services, including the screening and management of common illnesses, the distribution of medicines, and the management of side effects. These were also trained on surveillance, case definitions, and risk communication. For instance, in the DRC, health workers, especially those who had supported the Ebola response, were engaged to train RECOs for 3 days on surveillance, contact tracing, risk communication, the use of a thermo-flash for temperature screening, and the correct filling of the surveillance data collection forms. In Nigeria, VHWs were trained to conduct antenatal care, postnatal care, and referral of mothers to facilities for deliveries.

#### Development and dissemination of strategies, guidelines, and standard operating procedures

Countries developed strategies, simplified case definitions, and IEC materials for continuity of essential health services guidelines, which were translated into local languages to support CHWs deliver essential health services during COVID-19, surveillance, and risk communication. For instance, Uganda launched a national community engagement strategy for COVID-19 to strengthen the existing community health systems, ensure that infections are minimized, and enable prompt identification and response. The guidelines that were published by the MOH recommended task-shifting to community health workers for services that can be delivered at the community level [13].

#### Provision of mobile phones and tools

Community health workers were provided with phones, tools, registers for data collection, and follow-ups. In Uganda and Nigeria, some civil society organizations provided smartphones to community health workers to improve communication with health facilities and minimize the reduction of community health work due to the risk of infection. For instance, non-governmental organizations that support community health workers provided 4,300 community health workers with personal protective equipment [14]. LivingGoods Uganda also developed a mobile phone application that VHTs uploaded to their smartphones to facilitate adherence to the Ministry of Health guidance on preventing COVID-19 as they continued to provide care and treatment for malaria, diarrhea, and malaria for children under 5 years old and support mothers with antenatal and postnatal care [14].

*“Simple tools for data collection and reporting cases were developed*,* and the community was trained on how to use the tools both electronically and paper-based for those without a smart phone”* (Health Manager, Uganda).

#### Provision of personal protective equipment

Community health workers were provided with PPE such as face masks, gloves, and sanitizers to support the provision of essential health services and surveillance. For instance, in Uganda, the National Malaria Control Program reported that the Ministry of Health had facilitated VHTs with protective wear and was able to ensure the maintenance of community-based services such as indoor residual spraying for mosquito control and integrated community case management of childhood illnesses during the COVID-19 pandemic [15].

#### Community trust in CHWs

Several participants across all four countries felt that their pre-existing trust with the community members significantly aided them in asking questions, obtaining information, and educating communities about COVID-19 and its prevention. CHW could often relate to the population, speak their language, and successfully immerse themselves within the community. This was a great enabler for CHWs to support COVID-19 control and the continuity of the provision of non-COVID-19 services.

#### Other key facilitators

The development partners provided the community health workers with additional allowances. Community will and the availability of community structures such as community radios, as well as the provision of travel permits during curfews and lockdowns, facilitated sensitization.

## Discussion

In African countries where shortages, mal-distribution, absenteeism and poor motivation of health workers are rife, community health workers offer a potential additional resource to enhance systems resilience during emergencies [3]. This paper documents the roles played by the CHWs in the COVID-19 response and the barriers and facilitators in supporting COVID-19 response. Our findings show that CHWs supported the COVID-19 control and continuity of access to non-COVID-19 care. However, the CHWs faced several challenges, including transport constraints, inadequate PPE, a heavy workload, and low motivation due to low financial facilitation. A number of measures facilitated the CHWs’ work across countries. These included training, the provision of tools, including phones, guidelines, Information Education and Communication (IEC) materials and PPE.

In this study, the CHWs partly bridged the gap and supported access to services in the four countries through the direct provision of some of the services, mobilizing and sensitizing the community to access services. This finding is similar to what is reported in other African countries, including Ethiopia [16], Kenya, and Ghana [17]. Similarly, CHWs supported the continuity of essential health services during the 2014 Ebola outbreak in West Africa [18]. The CHWs are in direct contact with the community and have community trust. This gives them an advantage in mobilizing the community for services, easily reaching them with services, and conducting follow-ups. This implies that CHWs have the capacity to enhance adaptive resilience by filling health service gaps during epidemics, especially when health workers are overwhelmed. Therefore, there is a need to build the capacity of CHWs to support epidemic response and build health system resilience.

This study’s findings also show that CHWs supported the actual COVID-19 control in all four countries. This is similar to what was reported in Ethiopia, where CHWs supported risk communication, contact tracing. CHWs also made a similar contribution to the Marburg response in Uganda and the Ebola response in the DRC [16]. The ability of the CHWs to support COVID-19 control and other outbreaks is attributed to the fact that CHWs live within the communities and are trusted by these communities to give them accurate information about public health events [16, 19]. Thus, CHWs are an asset for social mobilization, addressing misinformation, fear, and stigma surrounding pandemics. This implies that these need to be leveraged early for an effective pandemic or outbreak response.

In Uganda, VHTs were instrumental in detecting cases in communities, contact tracing, and follow-up of patients during quarantine and home-based care. This role was also reported in Côte d’Ivoire [8] and Sierra Leone [7] during the 2014 Ebola epidemic, where CHWs conducted community surveillance activities and reported suspected Ebola cases to public health authorities. CHWs are a critical component of a community-based surveillance system. Therefore, having systems in place for CHWs to report symptoms or epidemiological patterns while performing their routine activities could enhance surveillance systems.

Despite the contribution of the CHWs to the COVID-19 response, they faced several challenges, including transport, supplies, and motivation challenges, among others. A related study by Olateju et al., (2022) in Nigeria reported similar challenges, including increased workload, lack of transport, and lack of PPE [20]. There is need for measures to address the challenges faced by CHWs to optimize their role in supporting the response and continuity of access to care. Furthermore, mitigation plans and contingency budgets that should include supplies and tools for CHWs.

Across the four countries, development partners supported community health systems to respond to COVID-19 and the support continuity of essential health services. Similarly, in Nigeria, financial incentives, PPE, and transport provision were the main improvements suggested by participants [20]. A previous study suggested that financial incentives increase motivation in CHWs [21]. Another study also discussed the need for adequate PPE provision for frontline workers [22]. Therefore, supportive mechanisms for CHWs need to be planned for and instituted to enable them to support health systems in responding to public health emergencies.

Our study findings indicate that the CHWs played an important role in ensuring that non COVID-19 health services continue while at the same time supporting COVID-19 control, contributing to the resilience of health systems [23]. In West Africa, CHWs also supported the continuity of essential health services, including maternal health services, during the Ebola outbreak and were key in building resilient health systems [7]. Drawing on these lessons will be critical for building resilient health systems for future pandemics and broader health systems.

### Limitations and strengths

The articles were screened based on title and abstract, which could have left out some relevant articles that missed CHW in the title and abstracts. This search was also limited to online documents published during the pandemic in the DRC, Nigeria, Senegal, and Uganda and could have missed out on some of the unpublished documents. However, we used a broad search strategy with a geographical focus to ensure that critical articles were captured and reviewed. We also included additional reports that the online search could have missed, and the findings in this paper were augmented by insights from key informants.

## Conclusion

This study has highlighted the critical roles CHWs played in supporting the COVID-19 response and the continuity of access to health care during the pandemic across the study countries. CHWs play a critical role in linking the community and the health system. Thus, to strengthen and build resilient health systems, investments in CHWs are very important. There is a need for sustainable strategies for addressing the key challenges affecting the optimal performance of CHWs across countries. CHWs need to be trained and continuously mentored, supported with the necessary equipment, supplies, and tools, and motivated through recognition for their efforts in supporting the response and control of pandemics and outbreaks.

### Electronic supplementary material

Below is the link to the electronic supplementary material.

Supplementary Material 1

## Data Availability

The Google data extraction excel data sheet and notes from the key informants are available on request from the corresponding Author.

## Abbreviations

CHW: Community Health Workers
COVID-19: The Corona Virus Disease 2019
DRC: Democratic Republic of Congo
EHS: Essential Health Service
IEC: Information Education and Communication
MoH: Ministry of Health
PPE: Personal Protective Equipment
RECO: Relais Communautaires
UNFPA: United Nations Populations Fund
UNICEF: United Nations Children Fund
USAID: United States Aids Agency for Development
VHT: Village Health Team
VHW: Village Health worker
WHO: World Health Organization
