# Supplementary material for: Leveraging community health workers for COVID-19 response in Democratic Republic of Congo, Nigeria, Senegal, and Uganda: roles, barriers, and facilitators
Source: BMC Prim Care. 2024 Jul 24;25:269. doi: 10.1186/s12875-024-02531-0 (PMC11267811; doi:10.1186/s12875-024-02531-0)
Supplement: Supplementary file 1 — Supplementary Material 1 [file 12875_2024_2531_MOESM1_ESM.pdf]

Consent form (English version)

Appendix III: Informed consent for Participants

Title: **COVID-19: Assessment of the COVID-19 Response in Eastern, Central and Western Africa**

Schools of Public Health in Nigeria, Uganda, DRC,Senegal and Ghana, are conducting a study across their respective African countries to assess, understand and document the (i) response to the COVID-19 pandemic in Africa and the outcomes in terms of COVID-19 control and (ii) continuity of essential non-COVID-19 services within the COVID-19 period. We also aim to document the positive or negative unintended consequences including socioeconomic and gender related vulnerability. The results from this study will inform the COVID-19 response and recovery as well as health system resilience to future disease outbreaks in Africa.

Risks from Being in the Study: Potential risks include: The risk that your views may become known to other people who have not participated in the interviews. We will minimize this by ensuring that only the individuals directly responsible for this study will have access to the interviews. Only authorized project personnel (approved by the study Principal Investigator) will have access to this information.

Benefits: You will not benefit personally from being in this study. However your responses and other study participants will be shared with national, regional players and actors to design measures which contributes to the COVID-19 response in sub-Saharan Africa.

Assurance of Confidentiality: Information collected from you will be kept confidential (secret) by Makerere University of Public Health to the full extent allowed by law. All data will be kept under password-protected computers to avoid unauthorized access to the data. Finally, your name will not be linked to your views; we will report about people's views in general and no attempt will be made to link the views to those who shared them.

Participation is Voluntary: Your participation in this study is completely voluntary. You are free to withdraw at any time or decline to participate in any or all components of the study. If you decide not to participate or withdraw from the study, you will still have access to the general health services offered.

Compensation: Participation in this study will be completely voluntary and no form of compensation will be provided.

Questions/Points of Contact: If you have any questions for me, about the study or the consent document, please ask before signing, and I will do my best to answer them. You will receive a copy of this consent form. If you have additional questions or if you need to discuss any other aspect of the study, you can contact: Dr. Rhoda Wanyenze, the Principal Investigator, based at the School of Public Health/Makerere University, Kampala (0772419762). If you ever have questions about your rights as a participant in this study, you should contact the acting

Chairperson of Higher degrees research committee, Dr. Joseph Kagayi P.O. Box 7072, Kampala or call 0393291397 OR the MakSPH IRB administrator Ms. Miria Nowamukama on 0778382007

If you agree to participate in this study kindly sign below (in duplicate)

__________________________ ____________________ ____________

Participant Name Participants Signature Date

__________________________ ____________________ ____________

Study Staff Study Staff Signature Date

Appendix IV: Key informant Interview Guide (English version)

Study Title: COVID-19: Assessment of the COVID-19 Response in Eastern, Central and Western Africa

Thematic Area: Role of Community Health workers in COVID 19 Response

Identifiers

i. Institution:
ii. Country:
iii. Office/Position:
iv. Duration in the position:
v. Age in complete years:
vi. Gender:
vii. Profession:

1. Please, what is your role in COVID -19 response?
2. What role did the Community health workers play in COVID 19 response
   a) What role did the CHWs play in COVID -19 control (probe for; surveillance, Isolation, Quarantine and Treatment, community mobilization, etc
   b) What role did the CHWs play in maintenance of essential health services? (probe for providing non COVID 19 services, community mobilization, referrals, etc)
3. What challenges did the community health workers face in supporting COVID 19 response /How did the pandemic affect CHWs role?

4. What innovations were put in place to enable the community health workers to support the COVID 19 response (probe for any support from government, development partners, that facilitated CHW work during COVID 19
5. What recommendations do you have for enhancing community health workers to support COVID 19 response and any future pandemics?
6. Any final thoughts?

Thanks

Formulaire de consentement (French)
Appendice III : Consentement éclairé des participants
Titre : COVID-19 : Évaluation de la réponse à la maladie en Afrique orientale, centrale et occidentale
Les écoles de santé publique du Nigéria, de l'Ouganda, de la République démocratique du Congo, du Sénégal et du Ghana procèdent à une étude dans leurs pays africains respectifs afin d'évaluer, de comprendre et de documenter (i) la réponse à la pandémie de COVID-19 en Afrique et les résultats en termes de contrôle du virus et (ii) la continuité des services essentiels non liés au virus dans la période du virus. Nous visons également à documenter les conséquences positives ou négatives non prévues, y compris la vulnérabilité socioéconomique et sexospécifique. Les résultats de cette étude donneront des informations sur la réponse et le rétablissement du COVID-19 ainsi que sur la résilience du système de santé aux futures épidémies de maladies en Afrique.
Risques liés à la participation à l'étude : Les risques potentiels comprennent: Le risque que vos vues puissent devenir connues d'autres personnes qui n'ont pas participé aux entrevues. Nous allons réduire cela en veillant à ce que seules les personnes directement responsables de cette étude aient accès aux entretiens. Seul le personnel du projet autorisé (approuvé par l'enquêteur principal de l'étude) aura accès à ces informations.
Avantages : Vous ne bénéficierez pas personnellement de votre participation à cette étude. Cependant, vos réponses et les autres participants à l'étude seront partagées avec les acteurs nationaux, régionaux et acteurs pour concevoir des mesures qui contribuent à la réponse au COVID-19 en Afrique subsaharienne.
Assurance de confidentialité : Les renseignements recueillis auprès de vous seront tenus confidentiels (secrètes) par Makerere University of Public Health dans toute la mesure permise par la loi. Toutes les données seront conservées sous des ordinateurs protégés par mot de passe pour éviter un accès non autorisé aux données. Enfin, votre nom ne sera pas lié à vos opinions; nous ferons état des opinions des gens en général et aucune tentative ne sera faite de lier les opinions à ceux qui les ont partagées.
La participation est volontaire : Votre participation à cette étude est entièrement volontaire.

Vous êtes libre de vous retirer à tout moment ou de refuser de participer à l'une ou l'autre des composantes de l'étude. Si vous décidez de ne pas participer ou de vous retirer de l'étude, vous aurez toujours accès aux services de santé généraux offerts. Indemnisation : La participation à cette étude sera entièrement volontaire et aucune forme de compensation ne sera fournie. **Questions/points de contact**: Si vous avez des questions pour moi, sur l'étude ou le document de consentement, s'il vous plaît demander avant de signer, et je ferai de mon mieux pour les répondre. Vous recevrez une copie de ce formulaire de consentement. Si vous avez des questions supplémentaires ou si vous avez besoin de discuter d'un autre aspect de l'étude, vous pouvez contacter: Dr. Rhoda Wanyenze, l'Investigateur Principal, basé à l'École de la santé publique / Université Makerere, Kampala (0772419762). Si vous avez des questions sur vos droits en tant que participant à cette étude, vous devriez contacter le Dr Joseph Kagayi P.O. Box 7072, Kampala ou appeler le 0393291397 OU l'administrateur de l'IRB de MakSPH Mme Miria Nowamukama au 0778382007

Si vous acceptez de participer à cette étude, veuillez signer ci-dessous (en duplicate)

________________________ ________________________________

Nom du participant Date de signature

________________________ ____

Date de signature du personnel d'étude

Appendice IV : Guide d'entretien de l'informateur clé (French)

Titre de l'étude : COVID-19 : Évaluation de la réponse à la maladie en Afrique orientale, centrale et occidentale

Domaine thématique : Rôle des travailleurs de la santé communautaire dans les identificateurs de la réponse au COVID 19

i. Institution: ii. Pays: iii. Office/Position: iv. Durée du poste: v. Âge dans les années complètes: vi. Genre: vii. Profession:

1. S'il vous plaît, quel est votre rôle dans la réponse au COVID-19?

2. Quel rôle ont joué les professionnels de la santé communautaires dans la lutte contre le COVID 19?

a) Quel rôle ont joué les CHW dans la lutte contre le COVID-19 (épreuve pour; surveillance, isolation, quarantaine et traitement, mobilisation communautaire, etc.)

b) Quel rôle ont joué les SGC dans le maintien des services de santé essentiels? (probe for providing non COVID 19 services, community mobilization, referrals, etc)

3. Quels sont les défis auxquels sont confrontés les travailleurs de la santé communautaire pour soutenir la réponse au COVID 19 / Comment la pandémie a-t-elle affecté le rôle des CHW?

4. Quelles innovations ont été mises en place pour permettre aux travailleurs de la santé communautaire de soutenir la réponse au COVID 19 (prouver tout soutien de la part des gouvernements, des partenaires de développement, qui a facilité le travail de la CHW pendant le Covid 19)

5. Quelles recommandations avez-vous pour renforcer les professionnels de la santé communautaires afin de soutenir la réponse au COVID 19 et les pandémies futures?

6. Avez-vous des réflexions finales?

Merci
